# Vibration and Noise in Magnetic Resonance Imaging of the Vocal Tract: Differences between Whole-Body and Open-Air Devices

**DOI:** 10.3390/s18041112

**Published:** 2018-04-05

**Authors:** Jiří Přibil, Anna Přibilová, Ivan Frollo

**Affiliations:** 1 Institute of Measurement Science, Slovak Academy of Sciences, 841 04 Bratislava, Slovak Republic; ivan.frollo@savba.sk; 2 Faculty of Electrical Engineering and Information Technology, Slovak University of Technology in Bratislava, 812 19 Bratislava, Slovak Republic; anna.pribilova@stuba.sk

**Keywords:** magnetic resonance imaging, acoustic noise, mechanical vibration

## Abstract

This article compares open-air and whole-body magnetic resonance imaging (MRI) equipment working with a weak magnetic field as regards the methods of its generation, spectral properties of mechanical vibration and acoustic noise produced by gradient coils during the scanning process, and the measured noise intensity. These devices are used for non-invasive MRI reconstruction of the human vocal tract during phonation with simultaneous speech recording. In this case, the vibration and noise have negative influence on quality of speech signal. Two basic measurement experiments were performed within the paper: mapping sound pressure levels in the MRI device vicinity and picking up vibration and noise signals in the MRI scanning area. Spectral characteristics of these signals are then analyzed statistically and compared visually and numerically.

## 1. Introduction

The magnetic resonance imaging (MRI) tomograph is basically a huge intelligent sensor used for biomedical purposes. Two different types of MRI equipment were analyzed and compared in the framework of this paper. They both work with a weak stationary magnetic field *B*_0_ up to 0.2 T but with totally different mechanical construction and different physical principle of this magnetic field creation. A pair of permanent magnets is usually incorporated in the open-air MRI device being normally used in clinical diagnostic practice for scanning smaller parts of human body such as a hand, a neck, a coxa, a knee, etc., or various biological tissues [1]. On the other hand, a resistive magnet containing a water-cooled multi-section coil is used for generation of a basic magnetic field in larger whole-body device enabling MR scans of more complex parts of the human body. Every MRI device consists of a gradient system to select *x*, *y*, and *z* slices of a tested subject. In the open-air MRI system, planar gradient coils [2] are mostly used to minimize space requirements. For the whole-body devices, there is typical use of cylindrical gradient coils distributed around the tube in which an examined person/object lies. There are also many differences in construction and practical realization of open-air and whole-body types of these devices. In spite of all the differences, both devices have in common undesirable production of significant mechanical pulses during execution of a scan sequence. Although magnetic translational forces and torques on diamagnetic and paramagnetic tissues are not of safety concern, this does not apply to acoustic noise as a result of rapid switching of large currents accompanied with rapid direction reversal of Lorenz forces [3]. The radiated acoustic noise can be measured by a microphone and its sound pressure level (SPL) can be mapped in the MRI neighborhood. The component frequencies of this acoustic noise fall into the standard audio frequency range, so it can be processed in the spectral domain and analyzed using methods similar to those of audio and speech signal analysis.

These MRI devices can also be successfully used for analysis of the human vocal tract structure and its dynamic shaping during speech production [4]. For this purpose, the speech signal must be recorded simultaneously in real time while the MR scan sequence is being executed [5]. The speech signal should be recorded with high signal-to-noise ratio (SNR), but an acoustic noise produced by the MRI gradient system degrades its quality [6]. Thus, noise reduction techniques must be applied to improve the SNR of the speech signal [7,8]. One group of enhancement methods is based on spectral subtraction of the estimated background noise [9]. However, noise estimation techniques based on statistical approaches are not able to track real noise variations; thereby they result in an artificial residual musical noise and a distorted speech [10]. Therefore, spectral properties of both vibration and noise generated by the gradient system of the MRI device must be analyzed with high precision so that the noise could be efficiently suppressed while preserving maximum quality of the processed speech signal [11].

The main motivation of this study was to measure and compare intensity, distribution, and spectral properties of mechanical vibration and acoustic noise produced by the low magnetic field MR imagers. As both types of investigated tomographs use the same physical principles for modulation of the basic magnetic field, we suppose comparable results of measured vibration and noise signals. These results can be generalized for next use, e.g., when direct measurement is difficult or practically impossible or undistorted values cannot be obtained. Hence, it is helpful that we can use results from the alternative type of MRI with the final aim to suppress negative influence of noise in the recorded speech signal while using a similar device. The original contribution of our paper lies in investigation and comparison of two low-field MRI devices with similar magnetic flux density differing in construction.

The study also describes measurement experiments performed in the scanning area and in the neighborhood of the MRI equipment. First, for both types of investigated MRI devices, mapping of the SPL was performed in their vicinity. The main experiment consisted of real-time recording of the vibration and noise signals which were subsequently off-line processed—the determined spectral features were statistically analyzed, and the obtained results were visually and numerically compared. Attenuation and reflection of the acoustic wave caused by the enclosing metal shielding cage, and influence of the mass of a tested person/object in the scanning area during execution of an MR scan sequence on the properties of vibration and noise signals were also discussed. Finally, the time delay between the vibration signal and the excitation impulse in the gradient coil from simultaneously recorded electrical excitation, vibration, and noise signals was analyzed and evaluated.

## 2. Subject and Methods

### 2.1. Differences in Construction of the Gradient System in the Open-Air and the Whole-Body MRI Equipment

Basic vibration and noise analysis was performed on the open-air MRI device [12] normally used in clinical diagnostic practice. This type of equipment has a stationary magnetic field with magnetic induction of 0.178 T produced by a pair of permanent magnets. The gradient system consists of 2 × 3 planar coils situated between the magnets and an RF receiving/transmitting coil with a tested object/subject. Different RF coils with cylindrical diameter not exceeding 18 cm are used for MR scans of a human knee, an arm, a leg, thin layers of botanical and zoological samples, or testing phantoms. Due to electromagnetic compatibility and reduction of possible RF signal interference, the whole MRI scanning equipment is located inside a metal cage. It is made of 2-mm thick steel plate with symmetrically placed holes of 2.5-mm diameter in 5-mm grid to eliminate electromagnetic field propagation to the surrounding space (control room with operator console, etc.). Such a perforated surface successfully attenuates low-frequency sound if its wavelength is much larger than perforation thickness and diameter [13,14]. The orifice together with the backing air cavity forms a Helmholtz resonator whose frequency of sound absorption depends on size of these acoustic elements [15]. Since volume of air behind the apertures (surrounding air in a room with a cage inside) is rather great, the Helmholtz resonance frequency is rather low, and this effect can be neglected. However, each flat part of the metal surface (between perforations) may reflect sound energy towards inside if the wavelength of the sound is much lower than the size of this flat part.

The situation is totally different when the whole-body MRI device is investigated. In this case, the gradient system is made up of six cylindrical coils. Size of the gradient coils is also greater, since the tube diameter must enable insertion of the patient’s bed with an examined person. In the case of an experimental whole-body MR imager TMR96 used in measurements for this study, the device works with a magnetic field *B*_0_ = 0.1 T created by a resistive water-cooled magnet with a diameter of 1414 mm and a length of 2240 mm. The active part of the equipment is enclosed in a shielding metal cage with the size of a small room (550 × 340 × 230 cm) made of 2-mm thin copper sheet with a smooth surface that is fully sealed except for four ventilation holes. For this reason, it is supposed to be a good acoustic reflector. On the other hand, although the pick-up sensors are arranged outside the scanning area to eliminate interaction with the working magnetic field, they are very close to the examined person lying inside the scan tube, so the effect of reflected acoustic wave superposition can be neglected in the recorded sound signal. More robust construction and greater mass of this device would inhibit its vibration. However, higher energy of the impulse current must be applied to select 3D coordinates of a tested subject, so stronger Lorentz forces [16] act in the gradient coil system. In the final effect, vibration and noise levels inside the scanning area are usually higher than those in the open-air MRI with planar gradient coils.

Preliminary performed experiments have shown that the produced vibration and acoustic noise are principally influenced by a mechanical load of a person lying in the scanning area of the open-air MRI machine [17] where the examined person lies directly on the plastic cover of the bottom gradient coil. The whole-body MRI contains a movable bed which is not directly connected with the gradient coils, but for larger volume of the sample inserted in larger gradient coils, higher electric current must flow through the gradient coils to perform equivalent change in the magnetic field to choose each of the *x*, *y*, *z* coordinates in the selected field of view (FOV) [18]. Higher energy used for generation of the vibration signal also has an effect on its spectral properties. From the acoustic point of view, the test person/sample/phantom placed on the patient’s bed changes the overall mass and stiffness of the whole scanning system including the gradient coil structure. These changed mechanical properties result in different vibration than in the case of the plate weighted by the mass of a tested person. It means that, first of all, the spectral properties of the picked-up vibration signal are changed depending on the applied mechanical weight.

### 2.2. Sensors for Measurement in a Weak Magnetic Field Environment

**Figure 1:**
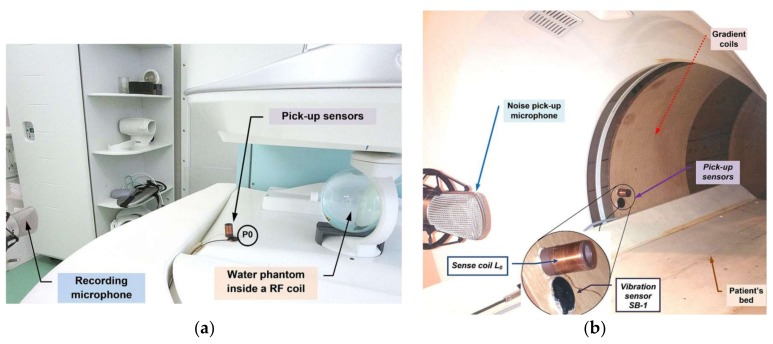
Photo of sensors placement for recording of vibration, noise, and electrical excitation signals in open-air and whole-body devices; (**a**) E-scan Esaote Opera with the spherical water phantom inside the knee RF coil; (**b**) MR imager TMR-96.

In general, the interaction with a stationary magnetic field *B*_0_ in the scanning area must be eliminated during measurement experiments to obtain MR images of sufficient quality without any artifacts. The same applies for measurement of noise SPL, excitation signal of the gradient coil system, and vibration and noise signals. In the case of MRI equipment working with a weak magnetic field (up to 0.2 T), the interaction problem can be solved by a proper choice of the arrangement where the measuring device (SPL meter and/or pick-up microphone) is located in an adequate distance from the noise signal source outside the magnetic field area. The choice of a suitable recording microphone was led by its good sensitivity and proper directional pickup pattern. Since the noise depends on the position of the measuring microphone, the directional pattern of the noise distribution in the MRI equipment neighborhood had to be mapped using optimal selection of the recording microphone position and parameters (distance from the central point of the MRI scanning area, direction angle, working height, type of the microphone pickup pattern). The sensors measuring vibration and electrical excitation signals must be placed inside the MRI scanning area where they are affected by a stationary magnetic field—see the documentary photo of measurement in and around the TMR96 device in Figure 1. In the scanning area, there is a high voltage generated by the excitation RF coil of the MRI device during execution of the MR sequence. This would result in large disturbance of a signal from the sensor or in damage of electronics integrated with the sensor. The vibration sensor with a piezoelectric transducer can be successfully used in these circumstances [11,12,17]. It is important that the sensor has good sensitivity and maximally flat frequency response. Its frequency range should cover harmonic frequencies of vibration and noise signals. These are concentrated in the low band due to frequency-limited gradient pulses [19], which is similar to the frequency range used for basic processing of speech signals. The above-mentioned requirements can be fulfilled by the sensor constructed for acoustic musical instrument pick up [20]. Finally, the sensing coil measuring the excitation signal must be designed with appropriate physical parameters (impedance, number of turns, mechanical construction, etc.) together with the input circuits for signal processing.

### 2.3. Features for Description of Vibration and Noise Signal Properties

For basic visual comparison of spectral properties of the recorded vibration or noise signals, a periodogram representing an estimate of a power spectral density (PSD) can be successfully used. Another useful graphical rendering is a spectrogram showing all PSD values in a time window moving through the whole analyzed signal.

Basic spectral properties of the vibration/noise are determined from the spectral envelope and subsequently histograms of spectral values are calculated and compared. MRI parameters of repetition time (TR) and echo time (TE) affect the dominant resonance *F*_V0_ (reciprocal of TR) and the secondary resonances *F*_V1,2_ (first two local maxima of the spectral envelope where its gradient changes from positive to negative or poles of the linear predictive coding transfer function). Spectral decrease (*S*_decrease_) is a parameter representing a degree of fall of the power spectrum. It can be calculated by a linear regression using the mean square method. A similar parameter is spectral tilt (*S*_tilt_) as an angle between a line connecting spectral envelope values at low and high frequencies and a horizontal line. Supplementary spectral features describe a shape of the power spectrum of the analyzed signal. Spectral centroid (*S*_centr_) determines a centre of gravity of the spectrum—the average frequency weighted by the values of the normalized energy of each frequency component in the spectrum. Spectral flatness (*S*_flat_) determining a degree of periodicity in the signal is calculated as a ratio of geometric and arithmetic means of the power spectrum. Shannon spectral entropy (*S*_entrop_) is a measure of randomness of the spectral probability density represented by normalized spectral components. Spectral spread (*S*_spread_) represents the dispersion of the power spectrum around its mean value.

In the last step, relationship between the primary electrical excitation of the gradient coils and the secondary generated acoustic noise is described. For this purpose, the time delay between these two signals must be analyzed. Indirect determination is based on statistical analysis of mutual positions of signal peaks of excitation and noise signals recorded in parallel. From the obtained distances, the histograms of percentage occurrence are calculated in dependence on the signal polarity and the maximum values of time delays *Td*_pos_, *Td*_neg_ are determined [12]. These two maxima are not equal for a non-planar surface of the lower cover of the gradient coil system. This means that vibration travels in two different paths between the point of its generation and the target position of the pick-up microphone. Then the final result is given by a median value of both maxima. The second method of time delay determination is based on direct calculation using formulae

(1) $$c=\sqrt{\frac{\gamma \cdot R\cdot T}{M}},\;\Delta t=\frac{D_{X0}}{c}=\frac{\Delta n}{f_{s}},$$

where *c* is velocity of sound propagation in the air at a given temperature, *γ* = 1.4 is air adiabatic constant, *R* = 8.31446 J K^−1^ mol^−1^ is universal gas constant, *T* [K] = *t* [°C] + 273.15 is thermodynamic temperature, *M* = 28.9647 × 10^−3^ kg mol^−1^ is air molar mass, *D_X_*_0_ is real distance between the noise microphone location and the excitation signal measuring point ∆*n* is corresponding number of samples, and *f_s_* is sampling frequency. These two approaches (direct and indirect) of time delay determination can be used to compare theoretical and real distances between the vibrating gradient coils and the noise sensor. However, this time delay involves superposition of a delay between the electrical excitation signal and the consequent vibration signal. Being a small delay, it is difficult to be determined in practice, but it causes an increase of the resulting theoretical distance *D_X_*.

## 3. Experiments and Results

This study encompasses three basic parts dealing with different comparisons in the area of MRI. The first part describes experiments for analysis of vibration and noise conditions in the scanning area and in the neighborhood of the open-air MRI equipment E-scan Opera by Esaote company Esaote S.p.A., Genoa, Italy [21], and the experimental whole-body experimental MR imager TMR96 device built at the Institute of Measurement Science (IMS) in Bratislava, using the Apollo (Tecmag Inc., Houston, TX, USA) console for control by the NTNMR ver. 1.4 software package [22]. Both investigated MRI devices are located at the IMS, in the laboratories of the department of imaging methods.

At first, different recording microphone positions and parameters (distance between the central point of the MRI scanning area and the microphone membrane, direction angle, working height, and microphone pickup pattern) are tested, and their effect on spectral properties of the recorded noise signal is analyzed. Next, the recorded electrical excitation, vibration, and noise signals are processed for visual comparison of spectrograms and periodograms. Then, basic and supplementary spectral features are statistically analyzed. Time delays between the electrical excitation impulses in the gradient coils and the subsequently generated mechanical vibration/acoustic noise are determined from the simultaneously picked-up signals. These delay times are visualized by histograms and occurrence density plots.

Two basic types of MR scan sequences called Spin Echo (SE) and Gradient Echo (GE) arising from physical principles of MRI [18] are used in the performed experiments. For real-time recording of the vibration signal, the piezoelectric SB-1 bass pickup was used. The acoustic noise was recorded by the 1′′ dual diaphragm condenser microphone B-2 PRO (by Behringer GmbH, Kirchardt, Germany) with final choice of a cardioid pickup pattern. For sensing the excitation signal, a special coil with an inductance *L*_0_ was designed and used—see documentary photos of measurement arrangement for both investigated MRI devices in Figure 1. The whole recording was performed by the Behringer Podcast Studio equipment used for connection to an external computer by the USB interface. A typical duration of the recorded signal was 30 s and for further signal processing the stationary parts lasting 15 s were selected using the sound editor program Sound Forge 8.0 by Sony Media Software, WI, USA. Subsequently, spectral properties of the recorded noise signals were analyzed. The temperature was always kept by air conditioning at 23 °C, giving the sound velocity of 345 m/s.

### 3.1. Mapping of Vibration and Noise Conditions in the Scanning Area of the Open-Air MRI Device

**Figure 2:**
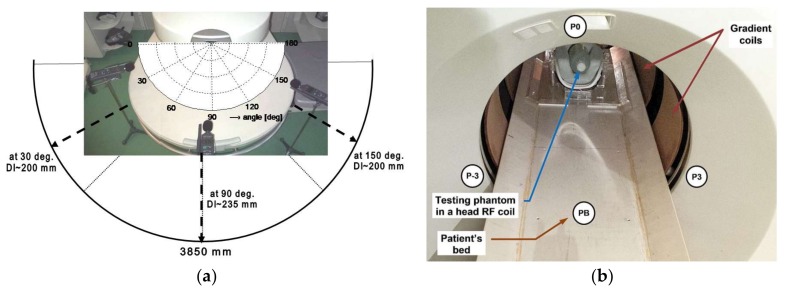
Arrangement of the noise and vibration measurements: in the open-air magnetic resonance imaging (MRI) device Opera together with principal angle diagram of the MRI scanning area; (**a**) sound pressure level (SPL) meter situated at 30°, 90°, and 150°; (**b**) in the whole-body imager TMR-96.

**Figure 3:**
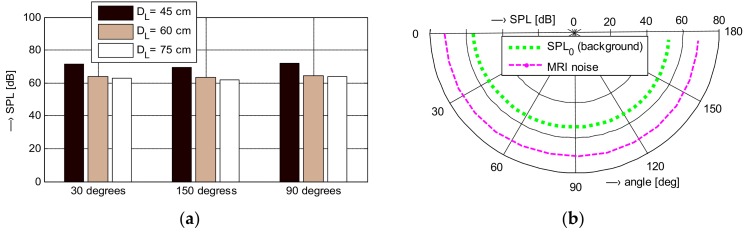
Visual comparison of obtained noise SPL values; (**a**) measured in the directions of 30°, 90°, and 150° at the distances of *D*_L_ = {45, 60, 75} cm; (**b**) measured directional patterns of the noise source and the background noise SPL_0_, *D*_L_ = 60 cm.

Basic mapping of vibration and noise conditions in the scanning area and in the neighborhood of the open-air MRI E-Scan Opera was performed within our previous research [12]. In the framework of the present study, two additional experiments were performed:

1. Measurement of the acoustic noise SPL in the MRI neighborhood in directions of 30°, 90°, and 150°—see the overview photo together with the principal angle diagram of the MRI scanning area in Figure 2a. Discrete MRI noise SPL values measured at distances of 45, 60, and 75 cm from the central point of the scanning area are shown in Figure 3a. The detailed measurement of the directional pattern of the acoustic noise SPL distribution was practically executed in the range of <0°~165°> in 15° steps (excluding the last one because of a patient bed at the position of 180°), at the distance of *D*_L_ = 60 cm from the MRI device central point—see the resulting diagram in Figure 3b. In both cases, the measurement was realized with the help of the sound level meter of the multi-function environment meter Lafayette DT 8820.
2. Parallel real-time recording of the signals from the electrical excitation, the vibration sensor, the microphone and/or the sound level meter. Comparison of both MRI devices in the form of histograms and occurrence density plots of basic and supplementary spectral properties together with the calculated time delays between the electrical excitation of the gradient coils and the subsequently generated noise can be found in Section 3.3.

The baseline measurement in the open-air device Opera was carried out during the execution of 3-D and Hi-Resolution (Hi-Res) sequences that are used for scanning of a human vocal tract [11,12,17]. In order to obtain results comparable with those for the whole-body MRI device, the parameters of used Hi-Res SE HF scan sequence were set to TE = 26 ms and TR = 500 ms. The auxiliary parameters were adjusted to 10 slices of 4-mm thickness and sagittal orientation, the spherical test phantom filled with doped water was inserted in the scanning RF knee coil. The sensors of electrical excitation and vibration signals were mounted directly on the lower plastic holder of the gradient coils in the direction of 45° at the point P0—see the arrangement photo in Figure 1a.

### 3.2. Analysis of Vibration and Noise Conditions of the Whole-Body MRI Equipment

The second collection of experiments was aimed at mapping noise conditions in the scanning area and in the vicinity of the experimental whole-body MR imager TMR-96 [23]. These experiments consist of

**Figure 4:**
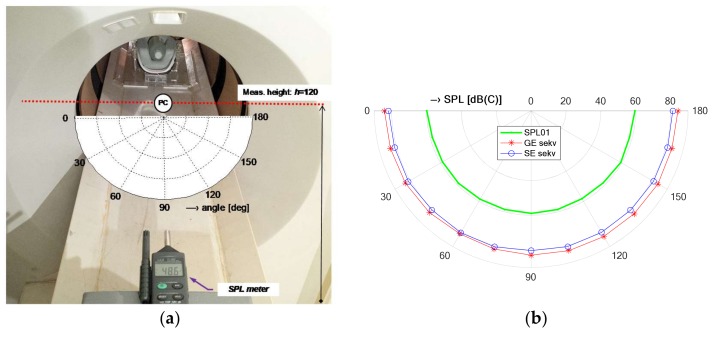
Arrangement of measurement of the acoustic noise SPL distribution in the vicinity of the TMR96 scanning tube; (**a**) SPL meter situated at the distance *D*_L_ = 45 cm from the scanning area center (point PC), in the height *h* = 120 cm above the floor level; (**b**) directional pattern for SE/GE sequences together with *SPL*_01_ values.

1. Measurement of the acoustic noise SPL in MRI neighborhood in the direction of 0° at three heights (2, 25, and 55 cm) above the patient’s bed level. Then, the SPL meter was located in ±120° (points P3 and P-3) at the height of 85 cm above the floor—see the arrangement photo in Figure 2b. The SPL meter was always placed at the distance of *D*_L_ = 60 cm from the front plastic panel to minimize interaction with the magnetic field. The measurement itself was carried out during the SE scan sequence with TE = 18 ms, TR = 400 ms under three noise conditions (obtained discrete noise *SPL* values are presented in Table 1).
  - *SPL*_00—_the background noise when all devices are stopped,
  - *SPL*_01—_the ventilators inside the copper cage are running,
  - *SPL_X_*_—_the scanning MR sequence is being executed with ventilation fans running.
2. The detailed measurement of the directional pattern of the acoustic noise SPL distribution in the MRI tube vicinity in the range of 0°~180° with 15° steps, at the distance of *D*_L_ = 45 cm from the MRI center (point PC) of the scanning area, in the high *h* = 120 cm above the floor level (25 cm above the patient’s bed)—see the arrangement photo in Figure 4a and the resulting diagram for GE/SE sequence (TE = 18 ms, TR = 400 ms) together with *SPL*_01_ curve in Figure 4b. In both cases, the measurement was realized with the help of the sound level meter of the multi-function environment meter Lafayette DT 8820.
3. Real-time recoding of the voltage signal from a piezoelectric transducer of the SB-1 sensor during execution of a chosen scan MR sequence (SE/GE type with different TE and TR parameter settings) and parallel recording of the electrical excitation signal (impulses from the MRI device gradient coil system) and/or the signals from the vibration sensor/pick-up microphone for time delay calculation and spectral properties comparison.

**Figure 5:**
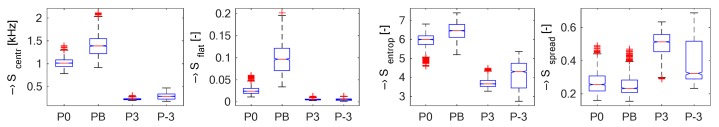
Box-plot of basic statistical parameters of supplementary spectral properties (centroid, flatness, entropy, spread) determined from the vibration signal picked up at different measuring positions {P0, PB, P3, P-3} during the SE sequence (TE = 18 ms, TR = 400 ms).

**Figure 6:**
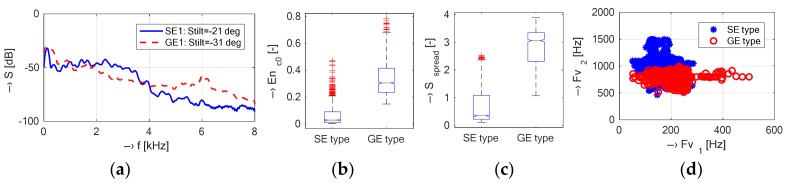
Visualization of differences of selected features of the recorded vibration signals; (**a**) spectral envelopes and calculated spectral tilts; (**b**) boxplot of basic statistical properties for *En*_c0_; (**c**) *S*_spread_; (**d**) mutual positions of *F*_v1_ and *F*_v2_ for SE/GE scan sequences with TE = 18 ms and TR = 500 ms.

The succession of sampling, resampling to 16 kHz, off-line signal processing, and analysis of spectral properties was similar to that in the open-air device. Here, the test phantom consists of a 1-liter plastic bottle filled with doped water [24] inside the head RF coil located on the patient’s bed in the middle of the MRI device scanning area. The second comparison experiment was focused on testing the influence of different locations of the vibration sensor and different scan sequences on spectral properties of the vibration signal. The succeeding analysis and comparison were aimed at:

- Mapping of vibration in different parts of the MRI device—the sensor mounted directly on the surface of the front plastic cover at the points P0, P3, P-3, and on the surface of the patient’s bed (PB). The numerical results of the basic spectral features can be seen in Table 2 and the box-plot statistics of the supplementary spectral properties in Figure 5.
- Determination of differences between two mostly used MR scan sequences of SE and GE types; the pick-up sensor at the P3 point—see the visualization of differences of the selected signal features in Figure 6.

### 3.3. Comparison of Spectral Properties of Vibration and Noise Signals Recorded in Open and Closed MRI Devices

The vibration and/or noise signals recorded in the open-air Opera and the whole-body TMR96 MRI devices using the test phantom placed in the RF coil were compared graphically and grouped for both types of devices. If not stated otherwise, the signals were taken at the position P0 during execution of the MR sequence Hi-Res SE 26 HF (TR = 400 ms) for the Opera MRI device and the position P3 using the SE1-18 (TR = 400 ms) for the TMR96 device. The processed signals were used to compare

**Figure 7:**
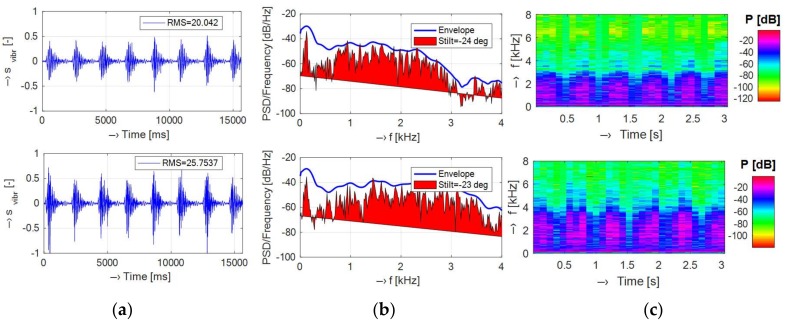
Visualization of basic spectral properties of recorded vibration signals; (**a**) stationary part of a normalized signal with its RMS value; (**b**) spectral density together with its envelope and calculated spectral tilt; (**c**) corresponding spectrograms for MRI Opera (upper set) and TMR96 (lower set).

**Figure 8:**
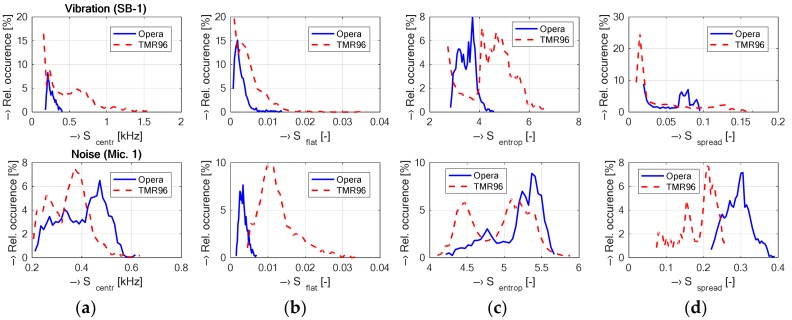
Histograms of supplementary spectral properties; (**a**) *S*_centr_; (**b**) *S*_flat_; (**c**) *S*_entrop_; (**d**) *S*_spread_, determined from picked-up vibration signals inside the Opera and TMR96 MRI devices.

**Figure 9:**
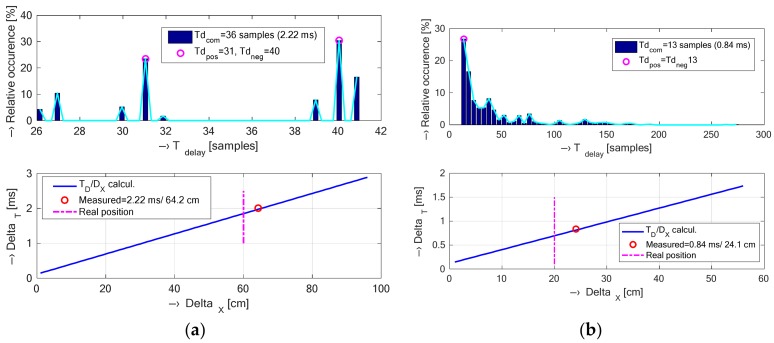
Histograms of evaluated time delays [samples] between electrical excitation and acoustic noise signals recorded in the MRI device (upper set), comparison of calculated and estimated mean values of time delays [ms] together with theoretical and real microphone distances (lower set); (**a**) for MRI Opera microphone Mic. 1 at a distance 60 cm and a direction 30°, sensing coil *L*_0_ at 45°; (**b**) for TMR96 Mic. 1 at a distance 16 cm from the patient’s bed position, *L*_0_ at P3, sequence SE1-18 (TR = 400 ms); *t* = 23 °C, *c* = 346 m/s.

- Basic spectral properties of vibration signals including spectral density, its envelope, spectral tilt, and spectrograms presented in the set of graphs in Figure 7;
- Histograms of supplementary spectral properties of vibration signals shown in Figure 8;
- Time delays between an electrical excitation signal and a generated acoustic noise (calculated from positive and negative pulses using the statistical method described in [12])—see the set of graphs in Figure 9.

## 4. Discussion and Conclusions

The measurements in the vicinity of the open-air MRI equipment E-scan Esaote Opera have shown that the maximum sound pressure level of about 72 dB(C) was achieved for the SPL meter located in the direction of 30°, the height of 85 cm (in the middle between the upper and the lower gradient coils), and at the distance of 45 cm, while the background noise SPL_0_ originating from the temperature stabilizer reached approximately 52 dB(C) measured in the time instant when no scan sequence was executed. Next, for three directions of 30°, 90°, and 150°, the noise SPL values measured with the examined person lying in the MRI scanning area were about 10 dB lower when compared with using the water phantom. The obtained noise SPL values were roughly inversely proportional to the effective weights of the male and female testing persons lying on the bottom plastic holder of the permanent magnet and gradient coils. On the other hand, the noise in the neighbourhood of the whole-body MRI device TMR96 achieved its maximum SPL of about 80 dB(C) using the SE scanning sequence and its minimum mean value of 62 dB(C) with no sequence running (the background noise generated mainly by the ventilators inside the cage) as documented by the numerical results in Table 1 and the detailed directional pattern in Figure 4b. In summary, it holds that the maximum SPL was observed for the sound level meter located at the point PB on the patient’s bed level and the minimum at the point P0. Evaluations of other authors are usually aimed at high-field MRI systems. Sound noise of various pulse sequences was compared for two whole-body MRI scanners by Cho et al. [25] with the rest value 79.5 dB(C) for the 1.5-T scanner and 68.6 dB(C) for the 2-T scanner. The highest sound pressure level of about 103 dB(C) was observed during the gradient echo sequence with TE = 4 ms, TR = 250 ms in the 1.5-T scanner and TE = 35 ms, TR = 100 ms in the 2-T scanner. Prince et al. [26] investigated acoustic noise in 15 MRI scanners giving a minimum of 82.5 dB(A) for a 0.23-T device using GE sequence, TE = 5 ms, TR= 525 ms and a maximum of 118.4 dB(A) for a 3-T device using the same sequence and TE, but TR = 3000 ms.

The vibration recording experiment was arranged to map basic points on the plastic cover as well as on the surface of the patient’s bed. In the case of the TMR96 device, the maximum vibration energy (expressed by RMS and/or from the first cepstral coefficient) was attained for the sensor placement on the patient’s bed almost at the top of the plastic cover (point PB)—see the mean values in Table 2. As regards the spectral features, the mean values of the vibration frequencies *F_V_*_1,2_ are the highest at P3 position (120°) and the lowest at the point P-3 (left bottom part of the plastic cover with the minimal vibration energy). The obtained results of the supplementary spectral properties are in good correlation with the basic ones—as documented by the visualization in Figure 5. Investigation of spectral differences between two mostly used MR scan sequences (SE/GE types) confirms our assumption that the GE sequence has more structured noise and the SE sequence generates more compact vibration with higher energy in the final effect, larger spread, and lower dispersion of *F_V_*_1,2_ frequencies as shown by the graphical results in Figure 6. Due to different construction of the open-air and the whole-body MRI devices, different software tools of their control systems, different types of used phantoms, etc., it was practically impossible to use the identical MR sequences. Only similar types of sequences with similar choice of basic parameters (TE, TR, orientation, etc.) could be applied. Consequently, the analyzed vibration signals had slightly different spectral features—see histograms in Figure 8. This general assumption was confirmed by the results presented in the form of spectrograms and periodograms. On the other hand, as documented by visualization of waveforms of the picked-up vibration signals in Figure 7a, the TMR96 device produces higher vibration levels with higher energy (signal_RMS_). This is in accordance with the basic physical law—greater scan volume in this device results in higher intensity of applied current in the gradient coils in spite of lower basic magnetic field (0.1 T vs. 0.178 T applied in the MRI Opera). As mentioned in the Section 2.1, the influence of different masses (volumes) in the scanning area on the intensity as well as on the spectral properties of the produced acoustic noise was analyzed in our previous research [17] using the MRI Opera device. In near future we would like to carry out similar experiments and measurements also with the TMR96 device.

In the last comparison experiment, we analyzed how the vibrations induced by the pulse current in the gradient coil travel through the holder of the MRI device, and how the actual position of the pick-up microphone corresponds with the one calculated from the determined time delay between the electrical excitation and the subsequently generated acoustic noise signals. As documented by the histogram in Figure 9a, the MRI device Opera has different time delay values determined from the positive and negative peaks of the compared signals. It means that there exist two maxima from which the final time delay was calculated as the median value. It is in agreement with the fact that the positive peaks have higher magnitude than the negative ones. This effect can be caused by the construction of the plastic cover of the gradient coils. The documentary photo in Figure 1a shows that the surface is not planar but slightly convexly curved. Hence, the mechanical force is different for positive and negative impulses originated from the gradient coils—in the case of negative ones the vibration acts against the force of the mechanical stiffness of the curved plate. In the measurement inside the TMR96 device, the sensors were mounted not on the plastic cover surface representing the front part of the whole MRI, but directly on the back part of the gradient coil surface. Though the measured surface was also curved, only one maximum of the time delay was observed in this case, see the histogram in Figure 9b. The obtained results of the backward comparison of the determined distance between the microphone picking up the noise and the origin of the vibration (sensor positions P0 for the MRI Opera and P3 for the TMR96) confirm our assumption that the distance calculated from the determined time delay values was always higher—see the bottom set of graphs in Figure 9. The detected increase of about 4–6 cm in the actual distance corresponds to the increase of the time interval by the delay during which the vibration is generated as a consequence of the excitation impulse in the gradient coil.

The results of the experiments will help to describe the process of the gradient coil electric excitation, the subsequent mechanical vibration, and the resulting acoustic noise generation in the MRI device scanning area and its vicinity. Additional measurement and analysis are necessary for better knowledge of these acoustic noise conditions. In the case of the MRI Opera, there is need for more information about the contribution of the upper gradient coil (and its plastic holder) to the resulting acoustic noise. Therefore, in near future we plan to perform parallel measurement of the vibration signal on the surface of both plastic holders. As regards the TMR96 device, the process of noise and vibration generation inside the scanning tube of the whole-body tomograph must be known. Thus, the measurement with the vibration sensor mounted in the place of the second and third gradient coils must be also performed for detailed mapping of the vibration in the whole 360° angle around the gradient coils. Critical parts (possible loose mounting to the main mass of the resistive magnet) can be found by this method, subsequently repaired, and/or some damping material might be inserted for mechanical suppression of the generated vibration and noise.

## Figures and Tables

**Table 1:** Measured *SPL* [dB(C)] at different positions.

Noise Condition/Measuring Position	at 0°		at 120°		at 120°	
	*h*_0_^1^ = 55 cm	*h*_0_^1^ = 25 cm	*h*_0_^1^ = 2 cm	*h*_1_^2^ = 85 cm		*h*_1_^2^ = 85 cm
*SPL*_00_ silent	47.9	47.7	47.5	47.4		47.5
*SPL*_01_ + ventilators	54.1	56.8	56.9	61.8		59.6
*SPL_X_* scan sequence	77	79.1	80.1	79.5		78.7

^1^ Height above the patient’s bed level. ^2^ Height above the floor.

**Table 2:** Mean values of basic spectral features of the recorded vibration signals ^1^.

Sensor Position/Feature	Signal_RMS_ (-)	En_c0_ (-)	*S*_tilt_ (°)	*F*_V1_ (Hz)	*F*_V2_ (Hz)
0° (TR = 400)	4.3	0.47	−22	429	1380
120° (TR = 400/500)	2.7/2.4	0.38/0.31	−1/−14	352/260	1105/1048
−120° (TR = 400)	2.5	0.27	−13	368	930
Patient’s bed (TR = 400)	7.9	0.95	3	398	1662

^1^ Used SpinEcho sequence with TE = 18 ms in all cases.

## References

[1] Wellard R.M., Ravasio J.P., Guesne S., Bell C., Oloyede A., Tevelen G., Pope J.M., Momot K.I. (2014). Simultaneous magnetic resonance imaging and consolidation measurement of articular cartilage. Sensors.

[2] He Z., He W., Wu J., Xu Z. (2017). The novel design of a single-sided MRI probe for assessing burn depth. Sensors.

[3] Panych L.P., Madore B. (2018). The physics of MRI safety. J. Magn. Reson. Imaging.

[4] Mainka A., Platzek I., Mattheus W., Fleischer M., Müller A.S. (2017). Three-dimensional vocal tract morphology based on multiple magnetic resonance images is highly reproducible during sustained phonation. J. Voice.

[5] Kuortti J., Malinen J., Ojalammi A. (2018). Post-processing speech recordings during MRI. Biomed. Signal Process. Control.

[6] Freitas A.C., Ruthven M., Boubertakh R., Miquel M.E. (2018). Real-time speech MRI: Commercial Cartesian and non-Cartesian sequences at 3T and feasibility of offline TGV reconstruction to visualise velopharyngeal motion. Phys. Med..

[7] Sun G., Li M., Rudd B.W., Lim T.C., Osterhage J., Fugate E.M., Lee J.H. (2015). Adaptive speech enhancement using directional microphone in a 4-T MRI scanner. Magn. Reson. Mater. Phys. Biol. Med..

[8] Vahanesa C., Reddy C.K., Panahi I.M. Improving quality and intelligibility of speech using single microphone for the broadband fMRI noise at low SNR. Proceedings of the IEEE International Conference of the Engineering in Medicine and Biology Society (EMBC).

[9] Han L., Shen Z., Fu C., Liu C. (2016). Design and implementation of sound searching robots in wireless sensor networks. Sensors.

[10] Ding H., Soon I.Y., Yeo C.K. (2010). Over-attenuated components regeneration for speech enhancement. IEEE Trans. Audio Speech Lang. Process..

[11] Přibil J., Přibilová A., Frollo I., Ahmed N. (2016). Analysis of acoustic noise and its suppression in speech recorded during scanning in the open-air MRI. Advances in Noise Analysis, Mitigation and Control.

[12] Přibil J., Přibilová A., Frollo I. (2014). Mapping and spectral analysis of acoustic vibration in the scanning area of the weak field magnetic resonance imager. J. Vib. Acoust. Trans. ASME.

[13] Tayong R., Dupont T., Leclaire P. (2011). Experimental investigation of holes interaction effect on the sound absorption coefficient of micro-perforated panels under high and medium sound levels. Appl. Acoust..

[14] Zhao X., Wang X., Yu Y. (2018). Enhancing low-frequency sound absorption of micro-perforated panel absorbers by combining parallel mechanical impedance. Appl. Acoust..

[15] Gai X.L., Xing T., Li X.H., Zhang B., Wang F., Cai Z.N., Han Y. (2017). Sound absorption of microperforated panel with L shape division cavity. Appl. Acoust..

[16] Moelker A., Wielopolski P.A., Pattynama M.T. (2003). Relationship between magnetic field strength and magnetic-resonance-related acoustic noise levels. Magn. Reson. Mater. Phys. Biol. Med..

[17] Přibil J., Přibilová A., Frollo I. (2016). Influence of the human body mass in the open-air MRI on acoustic noise spectrum. Acta IMEKO.

[18] Liang Z.P., Lauterbur P.C. (1999). Principles of Magnetic Resonance Imaging: A Signal Processing Perspective.

[19] Winkler S.A., Alejski A., Wade T., McKenzie C.A., Rutt B.K. (2017). On the accurate analysis of vibroacoustics in head insert gradient coils. Magn. Reson. Med..

[20] Fraden J. (2010). Handbook of Modern Sensors. Physics, Designs, and Applications.

[21] E-Scan Opera (2008). Image Quality and Sequences Manual. 830023522 Rev.

[22] TNMR Reference Manual, Hardware Reference Manual, DSPect User Guide. http://www.tecmag.com/support_contact/pulse_sequences/.

[23] Andris P., Dermek T., Frollo I. (2015). Simplified matching and tuning experimental receive coils for low-field NMR measurements. Measurement.

[24] Andris P., Frollo I. (2013). Asymmetric spin echo sequence and requirements on static magnetic field of NMR scanner. Measurement.

[25] Cho Z.H., Park S.H., Kim J.H., Chung S.C., Chung S.T., Chung J.Y., Moon C.W., Yi J.H., Sin C.H., Wong E.K. (1997). Analysis of acoustic noise in MRI. Magn. Reson. Imaging.

[26] Prince D.L., De Wilde J.P., Papadaki A.M., Curran J.S., Kitney R.I. (2001). Investigation of acoustic noise on 15 MRI scanners from 0.2 T to 3 T. J. Magn. Reson. Imaging.

